# Supplementary material for: Alzheimer’s Disease Related Biomarkers Were Associated with Amnestic Cognitive Impairment in Parkinson’s Disease: A Cross-Sectional Cohort Study
Source: Brain Sci. 2024 Aug 2;14(8):787. doi: 10.3390/brainsci14080787 (PMC11352303; doi:10.3390/brainsci14080787)
Supplement: Supplementary file 1 [file brainsci-14-00787-s001.zip › brainsci-3057804-supplementary.pdf]

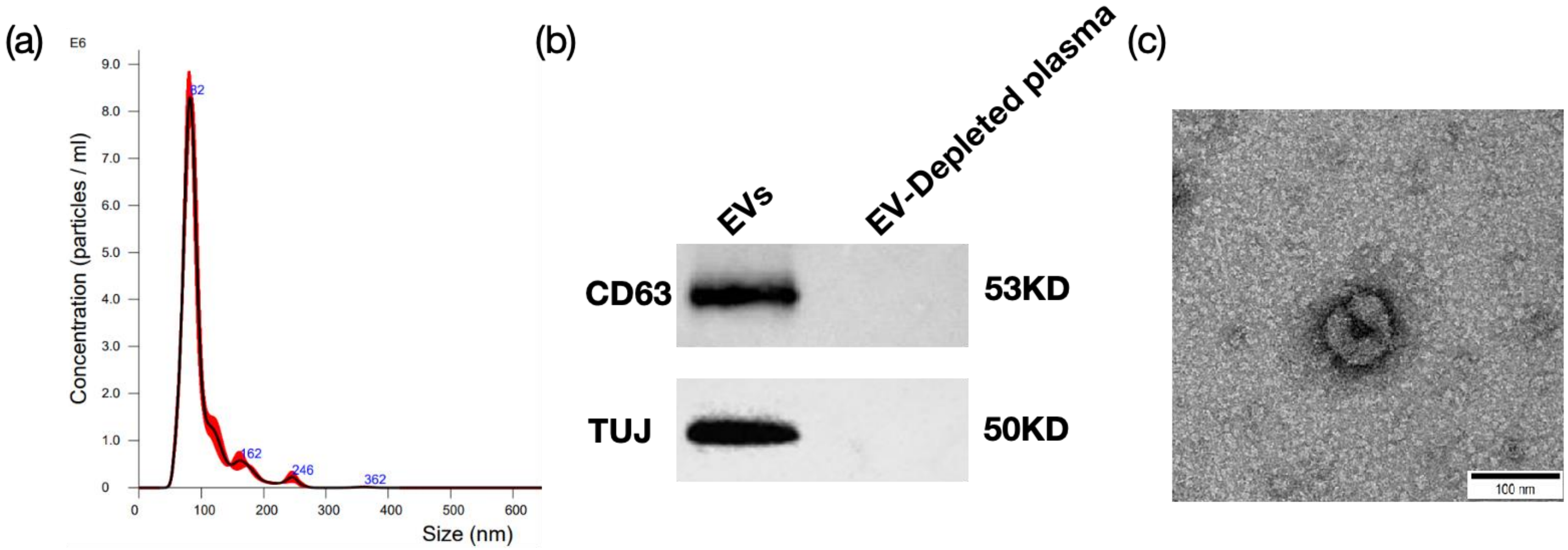

Figure S1 (a) The particle size analysis for NTA with a size distribution ranging from 50 to 200 nm and a predominant peak at 82 nm. (b) The Western Blot results indicating the detection of the EV universal marker CD63 and the neuronal EV surface marker TUJ1. (c) Electron microscopy image, revealing the typical cup-shaped morphology of EVs.

Abbreviations: NTA: nanoparticle tracking analysis; TUJ: Neuronal Class III β-Tubulin; EVs: extracellular vesicles; CD63: cluster of differentiation 63; EVs: extracellular vesicles.

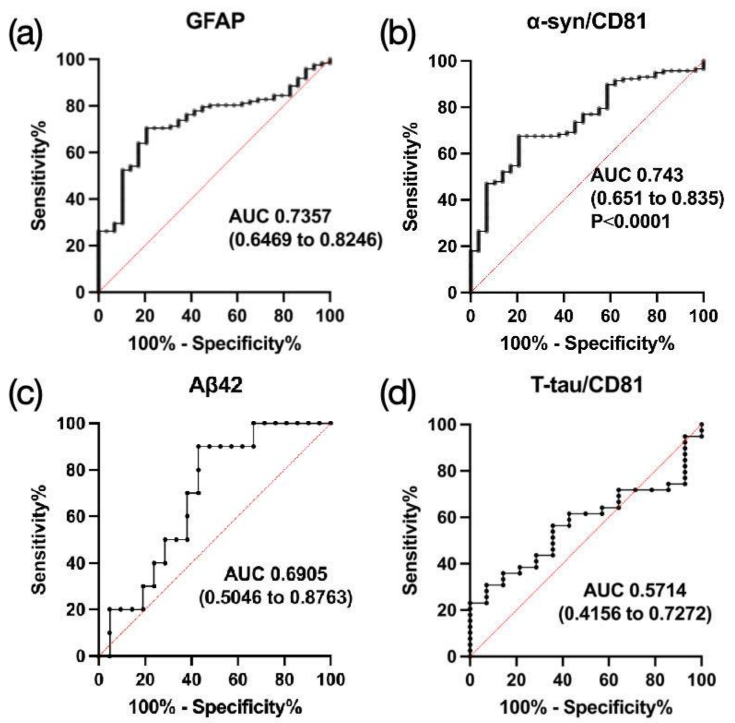

Figure S2 (a-b) ROC curve for discriminating the PD group from the HC group using plasma GFAP level and neuronal EV α-syn values as diagnostic parameters. (c) ROC curve for discriminating the Non-amnestic PD-NC group from the amnestic group using plasma Aβ42 level value as diagnostic parameters. (d) ROC curve for discriminating the Non-amnestic PD-MCI group from the amnestic group using neuronal EV T-tau/CD81 level value as diagnostic parameters. Abbreviations: AUC, area under the receiver operating characteristic curve.

Abbreviations: ROC: receiver operating characteristic; PD: Parkinson's disease; HC: healthy controls; EV: extracellular vesicle; PD-NC: PD with normal cognition; PD-MCI: PD with mild cognitive impairment; α-syn: α-synuclein; Aβ: beta-amyloid; T-tau: total tau; GFAP: glial fibrillary acidic protein; CD81: cluster of differentiation 81.

Supplementary Table S1. Comparison of biomarker levels between HCs and PD patients with different cognitive impairment.

Plasma/Neuronal EV Biomarkers (pg/ml)	HC (*n*=30)	PD (*n*=122)	*p*	PD-NC (*n*=31)	PD-MCI (*n*=56)	PDD (*n*=35)	*p*
α-syn	226.503±56.212	227.624±39.681	0.902	223.505±20.604	233.360±54.413	222.624±19.772	0.356
Aβ42	5.728±0.960	6.400±1.310	0.489	6.378±1.332	6.392±1.241	6.432±1.431	0.984
Aβ42/Aβ40	0.063±0.009	0.061±0.009	0.490	0.061±0.009	0.061±0.010	0.062±0.007	0.851
T-tau	1.277±1.078	1.057±0.526	0.110	1.294±0.595	1.052±0.509	0.863±0.451[e]	0.021
p-tau181	19.020±4.567	20.122±8.347	0.487	18.348±5.501	20.676±7.882	20.772±10.755	0.408
GFAP	70.415±19.539	99.583±42.135	<0.001	90.577±42.943	105.430±41.455	99.593±42.176	0.284
p-tau181/T-tau	27.276±15.685	32.304±13.283	0.775	16.494±8.591	26.130±16.776	55.558±18.570	0.068
α-syn/CD81	69.328±45.475	133.806±93.889	<0.001	136.739±99.432	116.937±78.007	159.341±108.93	0.119
Aβ42/CD81	1.240±0.572	1.186±0.958	0.774	1.092±0.738	1.335±1.206	1.027±0.579	0.313
Aβ42/Aβ40	0.311±0.065	0.322±0.115	0.611	0.285±0.057	0.342±0.146[b]	0.321±0.087	0.027
T-tau/CD81	0.219±0.209	0.270±0.264	0.333	0.276±0.233	0.301±0.329	0.214±0.138	0.324
p-tau181/CD81	4.672±3.942	5.257±7.073	0.680	6.531±2.448	5.645±4.595	3.525±1.618	0.216
GFAP/CD81	4.744±4.598	6.941±6.677	0.660	13.514±5.489	5.389±4.865	3.556±3.232	0.092
p-tau181/T-tau	26.770±19.944	28.601±12.725	0.829	32.668±16.259	31.663±16.743	20.386±10.853	0.556

Abbreviations: PD: Parkinson's disease; HC: healthy controls; EV: extracellular vesicle; PD-NC: PD with normal cognition; PD-MCI: PD with mild cognitive impairment; PDD: PD with dementia; α-syn: α-synuclein; Aβ: beta-amyloid; T-tau: total tau; p-tau181: phosphorylated tau181; GFAP: glial fibrillary acidic protein; CD81: cluster of differentiation 81.

[b]Compared with PD-NC, $p<0.05$; [e]Compared with PD-NC, $p<0.01$

Supplementary Table S2. Clinical Characteristics of Non-demented PD with and without amnestic syndrome.

	Non-amnestic PD-NC (*n*=21)	Amnestic PD-NC (*n*=10)	χ^2/p	Non-amnestic PD-MCI (*n*=14)	Amnestic PD-MCI (*n*=42)	χ^2/p
Age, years	60.620±11.681	56.800±4.566	0.230	65.290±6.742	64.790±8.008	0.835
Sex, male (%)	6 (28.6%)	4 (40.0%)	0.540	7 (50.0%)	26 (61.9%)	0.442
Education, years	14.330±2.082	14.000±1.764	0.666	12.210±2.486	12.790±3.025	0.526
Disease duration, years	5.350±4.344	3.890±2.713	0.363	4.360±2.274	4.800±4.191	0.706
Hoehn and Yahr score	2.470±0.841	2.430±1.512	0.923	2.310±1.182	2.950±1.050	0.071
MDS-UPDRSII score	6.900±2.713	8.220±8.715	0.536	7.150±3.412	9.850±6.937	0.184
MDS-UPDRSIII score	22.580±8.720	22.130±16.287	0.925	22.770±8.238	30.150±13.092	0.062
HAMD score	6.200±5.053	6.500±7.028	0.894	9.000±6.124	8.570±5.764	0.818
ESS score	2.740±2.330	4.000±3.496	0.254	4.540±4.521	3.790±3.440	0.526

Abbreviations: MDS-UPDRS II/III: Movement Disorder Society Unified Parkinson’s Disease Rating Scale, part II or part III; HAMD: Hamilton Rating Scale for Depression; ESS: Epworth Sleepiness Scale; PD: Parkinson’s Disease; PD-NC: PD with normal cognition; PD-MCI: PD with mild cognitive impairment.

Supplementary Table S3. ROC analyses of plasma Aβ42 level for predicting amnestic PD-NC versus non-amnestic PD-NC.

Plasma Biomarkers Non-amnestic PD-NC vs. amnestic PD-NC	Cutoff value	Sensitivity; %	Specificity; %	AUC (95% CI)	*P value*
Aβ42	6.510 pg/ml	57.1	90.0	0.6905 (0.5046-0.8763)	0.091

Abbreviations: PD: Parkinson’s Disease; PD-NC: PD with normal cognition; AUC, area under the receiver operating characteristic curve; CI, confidence interval.

Supplementary Table S4. ROC analyses of neuronal EV T-tau level for predicting amnestic PD-MCI versus non-amnestic PD-MCI

Neuronal EV biomarkers<br>Non-amnestic PD-MCI vs. amnestic PD-MCI	Cutoff value	Sensitivity;<br>%	Specificity;<br>%	AUC<br>(95% CI)	*P value*
T-tau/CD81	0.315	30.8	92.9	0.5714 (0.4156-0.7272)	0.431

Abbreviations: ROC: receiver operating characteristic; EV: extracellular vesicle; PD: Parkinson's Disease; PD-MCI: PD with mild cognitive impairment; AUC, area under the receiver operating characteristic curve; CI, confidence interval.

Supplementary Table S5. Correlation between clinical characteristics and plasma biomarker levels

	α-syn		Aβ42		Aβ42/Aβ40		T-tau		p-tau181		GFAP	
	t	*p*	t	*p*	t	*p*	t	*p*	t	*p*	t	*p*
Age	-0.136	0.141	0.447	0.656	-4.486	<0.001	0.949	0.345	1.498	0.138	5.729	<0.001
Sex	-0.064	0.488	0.406	0.686	-0.642	0.523	-1.307	0.195	-1.006	0.318	1.255	0.213
Education	0.083	0.367	1.245	0.217	-0.086	0.932	-1.202	0.233	-0.328	0.744	0.844	0.401
Disease duration	0.020	0.831	-1.586-	0.117	-0.665	0.508	0.043	0.966	-1.078	0.285	0.373	0.710
HY	0.122	0.214	0.937	0.351	1.891	0.062	0.521	0.604	0.425	0.672	-0.704	0.484
UPDRSII	0.184	0.052	-0.496	0.621	-1.080	0.283	0.079	0.937	1.373	0.174	0.317	0.752
UPDRSIII	0.136	0.161	-0.244	0.808	-0.146	0.885	-1.857	0.067	-0.206	0.837	0.970	0.335
HAMD	-0.018	0.849	-1.158	0.250	-1.066	0.289	-1.143	0.256	0.599	0.551	1.014	0.314
ESS	-0.022	0.820	-0.436	0.664	0.072	0.943	-0.620	0.537	2.042	0.045	0.962	0.339

Abbreviations: MDS-UPDRS II/III: Movement Disorder Society Unified Parkinson's Disease Rating Scale, part II or part III; HAMD: Hamilton Rating Scale for Depression; ESS: Epworth Sleepiness Scale; α-syn: α-synuclein; Aβ: beta-amyloid; T-tau: total tau; p-tau181: phosphorylated tau181; GFAP: glial fibrillary acidic protein.

Supplementary Table S6. Correlation between clinical characteristics and neuronal EV biomarker levels

	α-syn/CD81		Aβ42/CD81		Aβ42/Aβ40		T-tau/CD81		p-tau181/CD81		GFAP/CD81	
	t	*p*	t	*p*	t	*p*	t	*p*	t	*p*	t	*p*
Age	-0.136	0.145	1.745	0.085	1.022	0.310	0.734	0.465	-0.243	0.808	-0.305	0.761
Sex	-0.212	0.720	0.968	0.336	-0.994	0.324	2.328	0.023	-0.485	0.629	-0.398	0.692
Education	0.885	0.833	1.068	0.289	-1.188	0.239	1.106	0.272	0.729	0.469	1.044	0.300
Disease duration	0.439	0.379	-1.263	0.211	-0.394	0.695	-0.655	0.515	-0.547	0.586	-0.976	0.332
HY	-0.788	0.662	2.245	0.028	-0.292	0.771	1.307	0.195	0.945	0.347	0.763	0.448
UPDRSII	2.254	0.433	0.012	0.990	-0.240	0.811	-1.347	0.182	0.731	0.467	0.970	0.335
UPDRSIII	0.031	0.027	-0.199	0.842	1.317	0.192	0.852	0.397	0.325	0.746	0.278	0.782
HAMD	0.528	0.710	-0.702	0.485	0.488	0.627	1.063	0.291	-0.079	0.937	-0.387	0.700
ESS	0.924	0.358	1.299	0.198	-0.021	0.983	-0.201	0.841	0.018	0.985	-0.717	0.476

Abbreviations: EV: extracellular vesicle; MDS-UPDRS II/III: Movement Disorder Society Unified Parkinson's Disease Rating Scale, part II or part III; HAMD: Hamilton Rating Scale for Depression; ESS: Epworth Sleepiness Scale; α-syn: α-synuclein; Aβ: beta-amyloid; T-tau: total tau; p-tau181: phosphorylated tau181; GFAP: glial fibrillary acidic protein; CD81: cluster of differentiation 81.
